# Modulation of Biofilm Formation and Permeability *in* Streptococcus mutans during Exposure To Zinc Acetate

**DOI:** 10.1128/spectrum.02527-22

**Published:** 2023-02-21

**Authors:** Kara M. Buzza, Alain Pluen, Christopher Doherty, Tanaporn Cheesapcharoen, Gurdeep Singh, Ruth G. Ledder, Prem K. Sreenivasan, Andrew J. McBain

**Affiliations:** a Division of Pharmacy and Optometry, School of Health Sciences, Faculty of Biology, Medicine and Health, The University of Manchester, Manchester, United Kingdom; b HITLAB, New York, New York, USA; c Department of Oral Biology, Rutgers School of Dental Medicine, Newark, New Jersey, USA; University of Florida

**Keywords:** dental, oral, plaque, *S. mutans*, zinc, antimicrobial, biofilm, permeability, toothpaste

## Abstract

The penetration of biofilms by antimicrobials is a potential limiting factor in biofilm control. This is relevant to oral health, as compounds that are used to control microbial growth and activities could also affect the permeability of dental plaque biofilm with secondary effects on biofilm tolerance. We investigated the effects of zinc salts on the permeability of Streptococcus mutans biofilms. Biofilms were grown with low concentrations of zinc acetate (ZA), and a transwell transportation assay was applied to test biofilm permeability in an apical-basolateral direction. Crystal violet assays and total viable counts were used to quantify the biofilm formation and viability, respectively, and short time frame diffusion rates within microcolonies were determined using spatial intensity distribution analysis (SpIDA). While the diffusion rates within biofilm microcolonies were not significantly altered, exposure to ZA significantly increased the overall permeability of S. mutans biofilms (*P* < 0.05) through decreased biofilm formation, particularly at concentrations above 0.3 mg/mL. Transport was significantly lower through biofilms grown in high sucrose conditions.

**IMPORTANCE** Zinc salts are added to dentifrices to improve oral hygiene through the control of dental plaque. We describe a method for determining biofilm permeability and show a moderate inhibitory effect of zinc acetate on biofilm formation, and that this inhibitory effect is associated with increases in overall biofilm permeability.

## INTRODUCTION

Oral health is an important part of overall wellbeing (1). Caries (2) and gum disease (3) affect a large proportion of the global population (4). In the NHS UK Adult Dental Health Survey (5), it was reported that only 10 percent of people had excellent oral health (≥21 natural teeth, of which ≥18 are untreated, as well as no calculus, bleeding, pocketing, attachment loss, or decay). Estimates suggest that 90% of all adults will develop gingivitis at least once (1, 6), and untreated dental caries in permanent teeth remains the most common health condition, globally (7). Caries rates remain high in the Western world, despite good access to dental care services, largely due to a high sugar intake (8). Dietary sugars can be broken down by oral bacteria to form acidic fermentation products that can result in the formation of carious lesions through the erosion of the tooth enamel, and the subsequent exposure of dentine (9). As one of the most acidogenic species in the mouth, Streptococcus mutans is a key cariogenic bacterium (10) that has been identified as a crucial player in the ecological plaque hypothesis (11). The ability of *S. mutans* to form persistent biofilms in the oral cavity makes it an important target for oral health care measures (12).

Guidelines for maintaining oral health continue to be largely based on the mechanical and chemical removal of bacteria present in the oral cavity, which can form complex and resilient multispecies dental plaque biofilms, in particular, on the hard surfaces of the mouth (teeth), both above and below the gum line (13, 14).

The current advice is to brush twice a day for 2 min with a fluoridated dentifrice (15). However, poor adherence is a problem, with some studies suggesting that while 75% of UK adults report brushing twice a day (16), this may be an overestimate, with brushing times also being shorter than recommended (17). In a study of 14 volunteers, only 34% of brushing events recorded in 2 months complied with guidelines (120 s of brushing time), and this decreased to 24% over the next 6 months (17). With the aim of combatting this problem and to further aid in the removal of oral biofilms, antimicrobial agents are commonly added to fluoridated dentifrices. One such example is salts of the trace metal zinc, which has been shown to have antimicrobial properties (18) and may also inhibit acid production (19). For example, zinc oxide (ZnO) nanoparticles have been tested against a range of bacteria, and their effects on the bacterial cell membrane and subsequent cytoplasmic leakage have been documented by scanning and transmission electron microscopy in Gram-negative foodborne pathogens, including Escherichia coli and Campylobacter jejuni (20, 21). These effects are in part due to the antimicrobial properties of Zn^2+^ ions, which have been demonstrated against several oral pathogens (22). Furthermore, the bacteriostatic properties of Zn^2+^ against oral streptococci have been assessed (23). The effectiveness of dentifrices containing zinc with (24) and without (25, 26) other active ingredients has been investigated in various clinical studies to show antibacterial effects on dental plaque, and, in recent studies, reductions in salivary SARS-CoV-2 viral loads (27).

Biofilms, including dental plaque, are differentiated from their planktonic counterparts by the presence of an extracellular matrix, which can constitute up to 90% of the biofilm dry weight (as previously reviewed) (28). Polysaccharides are an important matrix component, but proteins, lipids, and extracellular nucleic acids (29, 30) are also present (30). Bacteria in biofilms generally display tolerance to antimicrobial compounds that is several orders of magnitude greater than those of their planktonic counterparts, and this tolerance occurs through several protective features afforded by the matrix, which include a reduction in the permeation rate and, thus, the concentration of antimicrobials reaching the bacteria (31). The mechanisms of this permeation rate reduction vary, depending on both the biofilm and the physicochemical properties of the compound in question, but they may include trapping the compound in the matrix, electrostatic or hydrophobic interactions resulting in retarded diffusion, and a reduced ability to penetrate areas other than the biofilm water channels, which thereby limits access to microbial cells, potentially resulting in subtherapeutic concentrations (31, 32). Increasing the permeability of oral biofilms to antimicrobial agents may therefore increase the anti-biofilm efficacy of the primary compounds and also that of secondary plaque control agents. This is a relatively little-studied area of oral microbial control. Therefore, we have investigated the viability and permeability of S. mutans biofilms that were grown in the presence or absence of low concentrations of zinc acetate, using both an adaption of the transwell transportation assay and spatial intensity distribution analysis (SpIDA) of a time-lapse image series that captured the localized diffusion of a fluorescent tracer through microcolonies via a confocal laser scanning microscope.

## RESULTS

### Effects of zinc on bacterial growth and biofilm formation.

**FIG 1:**
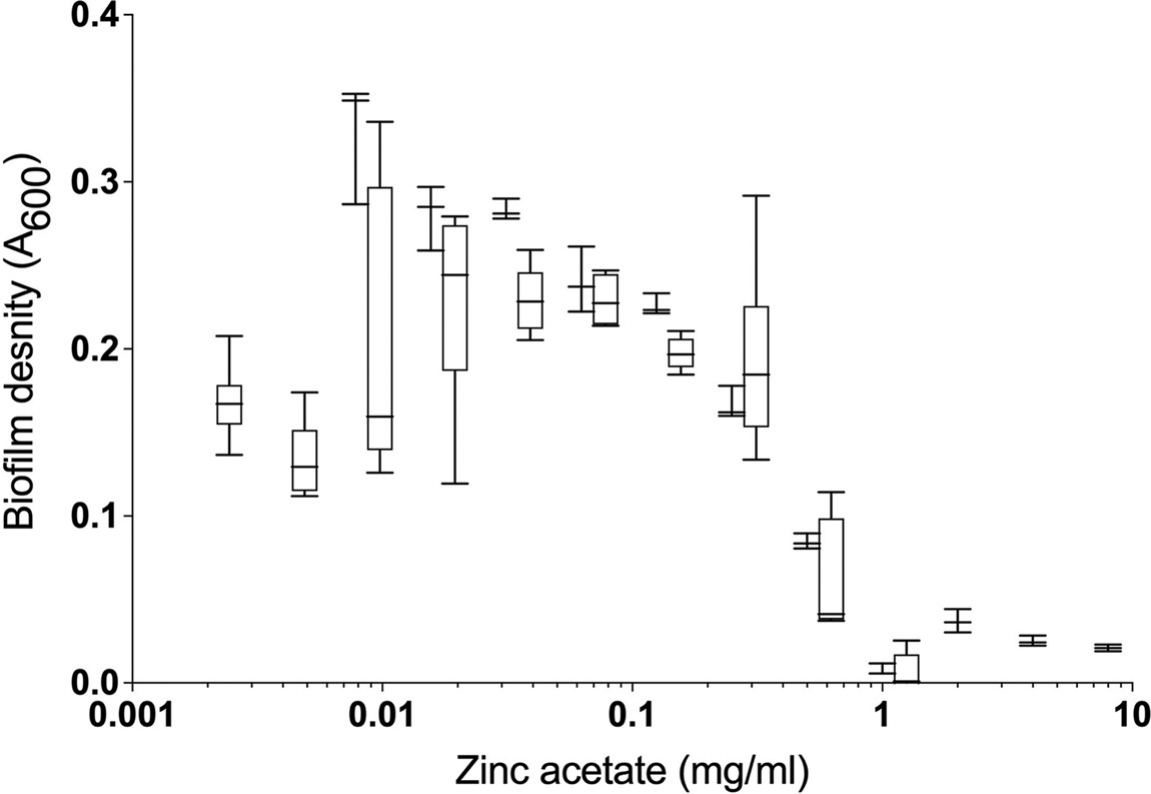
Biofilm formation of S. mutans in the presence of zinc acetate (0.0025 to 8.0 mg/mL) was determined using a crystal violet assay. The error bars show the standard deviation, which is representative of three biological replicates.

The minimum inhibitory concentration (MIC) of ZA against S. mutans was 1.0 (± 0.0) mg/mL. Crystal violet assays were used to measure the amount of biofilm formed in microtiter plate wells containing serial dilutions of ZA. The results, shown in Fig. 1, indicate the inhibition of biofilm formation at concentrations greater than 1 mg/mL. There was a steep increase in biofilm formation following treatment by the addition of ZA concentrations that ranged from 0.3125 mg/mL to 1 mg/mL, after which the amount of biofilm formed stabilized and no longer significantly differed from that of the negative control (*P* > 0.05, Mann-Whitney test), apart from at 0.0049 mg/mL (*P* = 0.009).

### Apical-basolateral permeability of biofilms measured using the transwell transportation assay.

**FIG 2:**
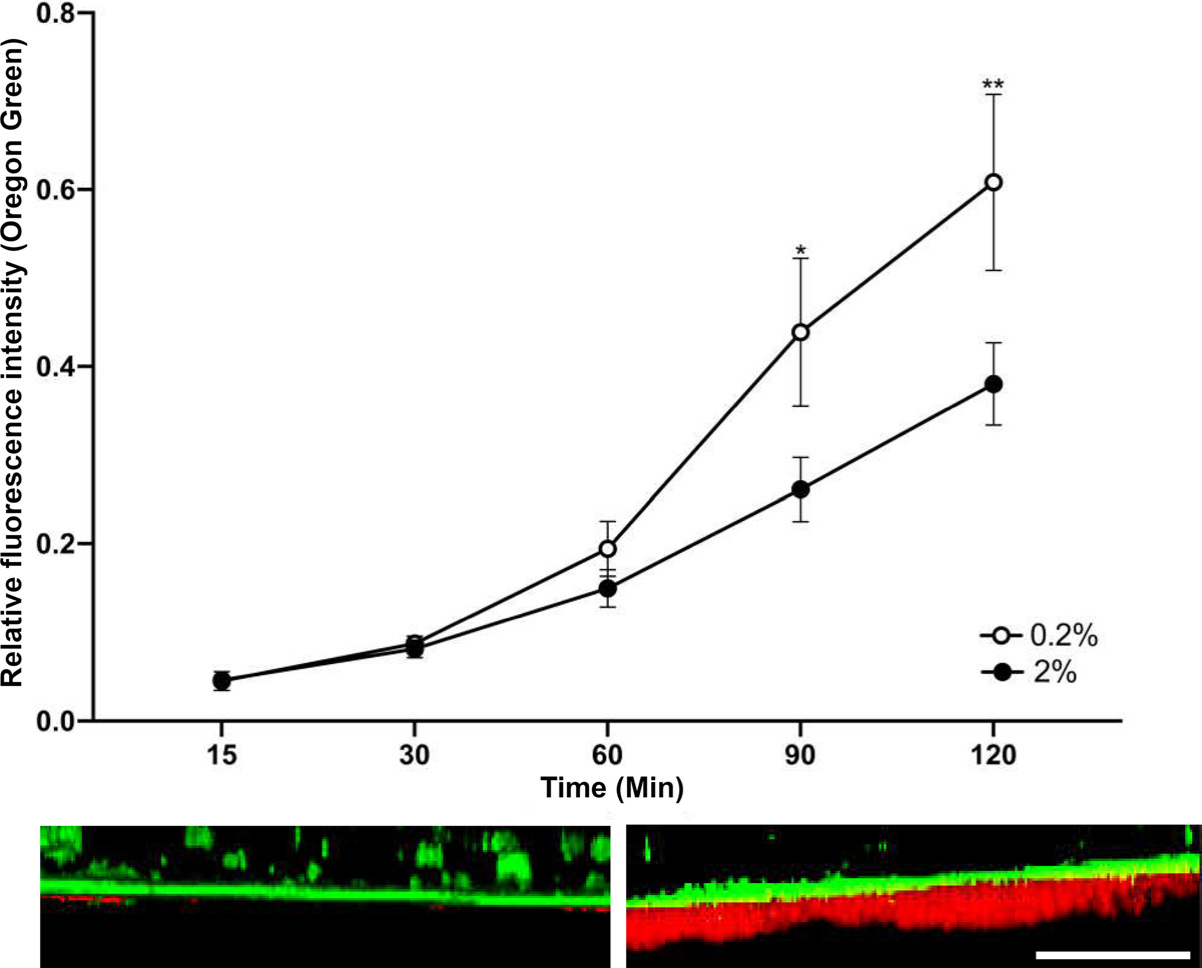
Biofilms were grown on transwells in the presence of 0.2% or 2% sucrose. Biofilms were stained with Toto -1 iodide and Syto 60, demonstrating a thicker layer of extracellular DNA, a biofilm component, and greater bacterial intracellular staining when grown in the presence of 2% sucrose (right), in comparison to 0.2% sucrose (left). Scale bar = 50 μm.

**FIG 3:**
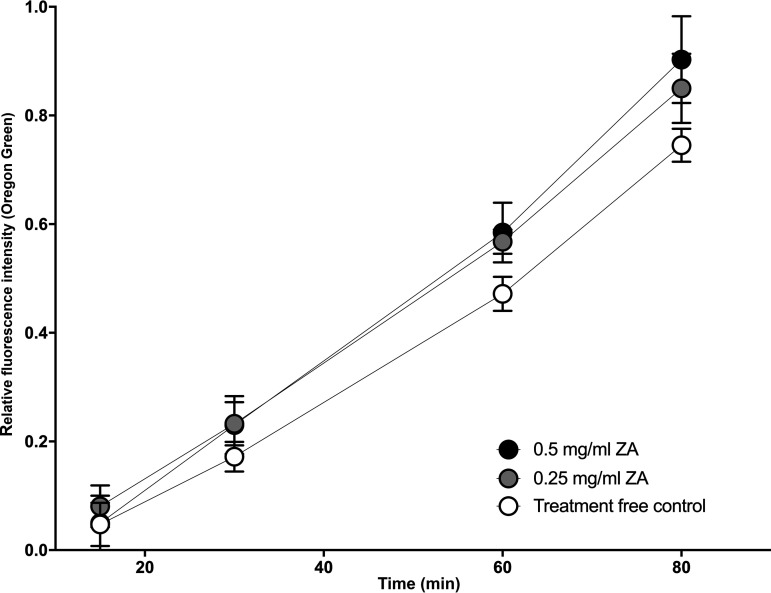
The permeability of the S. mutans biofilms was determined by adapting a transwell transportation assay and measuring the amount of a fluorescent tracer, Oregon Green (OG), which travelled through the biofilm in an apical-basolateral direction. The results were normalized and are shown as the relative concentration of OG over time. Biofilms were grown in the absence or presence of 25% or 50% of the MIC of zinc acetate (0.25 and 0.5 mg/mL, respectively) for 5 d before the testing and OG measurements were performed at 15, 30, 60, and 90 min. The error bars represent the standard deviation based on six biological replicates. *, *P* < 0.05 for 0.5 mg/mL ZA versus a control, #, *P* < 0.05 for 0.25 mg/mL ZA versus a control.

The diffusion of Oregon Green (OG) through mature S. mutans biofilms grown in the presence or absence of sublethal concentrations of ZA was measured using an adaptation of the transwell transportation assay. In validation experiments, biofilms that were grown in medium supplemented with 0.2% (w/v) sucrose were markedly thinner than those grown with 2% (w/v) sucrose, and OG transport was significantly retarded in the thicker biofilms (Fig. 2). The amount of OG that travelled through each S. mutans biofilm was normalized for the two separate experiments to adjust for any differences in the concentration of the OG solution used, and the combined results are shown over time in Fig. 3. The treatments were compared using a repeated-measures analysis of variance (ANOVA) with Tukey’s *post hoc* test for multiple comparisons, which showed a significant difference between both zinc-treated groups (0.25 and 0.5 mg/mL) and the treatment-free control (*P* < 0.05). The groups were also compared using unpaired *t* tests for each time point, showing that both zinc treatments gave significantly higher OG concentrations than did the control at 30 and 60 min. (*P* < 0.05). Only 0.5 mg/mL ZA was significantly different from the control at 90 min. (*P* < 0.05). There were no significant differences observed between the ZA treatment groups. The data are representative of six biological replicates.

### Correlation between biofilm viable counts and apical-basolateral permeability.

**FIG 4:**
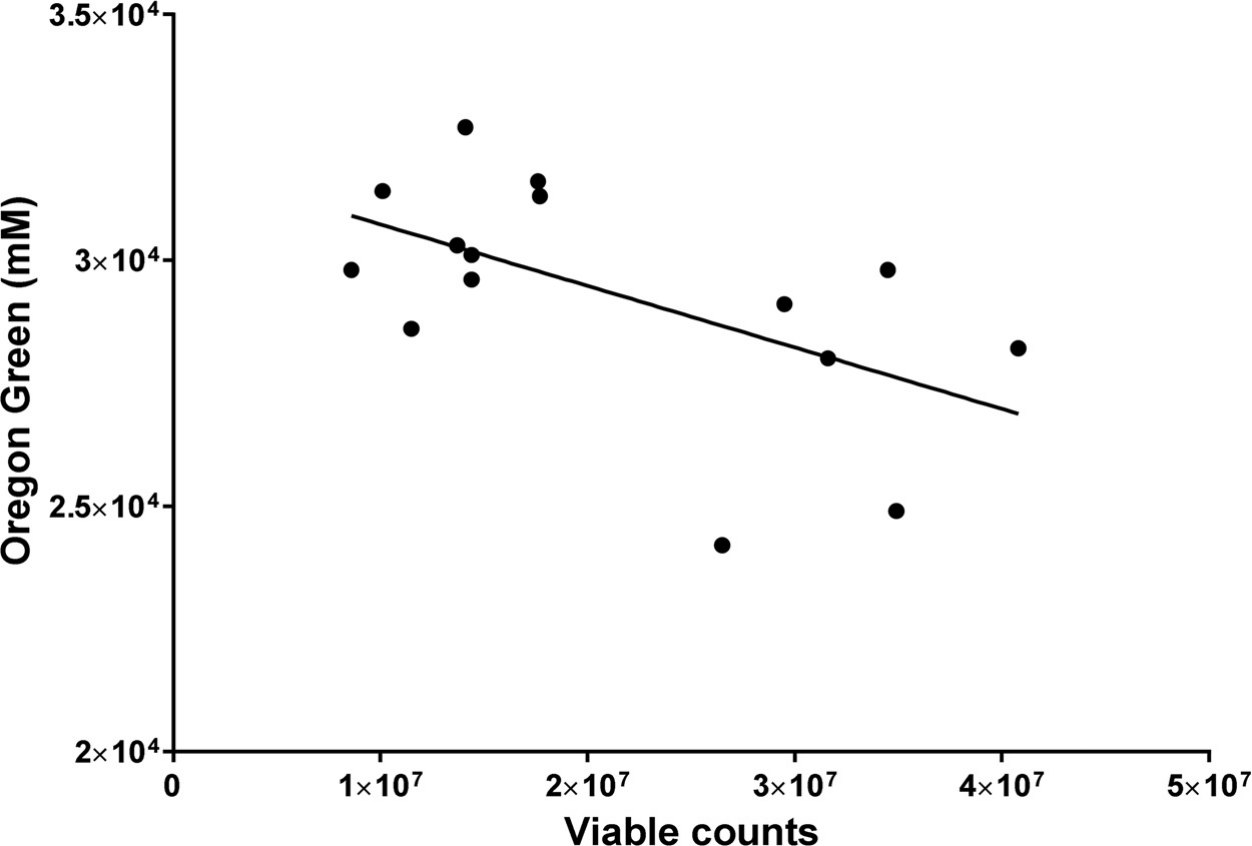
The correlation between biofilm permeability and viable count data is shown as a scatterplot with a linear trend line. Pearson’s correlation coefficient was calculated and gave a moderate negative correlation of −0.57 (*P* < 0.05).

The viability of biofilms grown in the absence or presence of different concentrations of ZA was determined using total viable counts, and the correlation between these and the permeability of each biofilm was determined by calculating Pearson’s correlation coefficient, which was −0.57 (*P* < 0.05). This represents a moderate but statistically significant negative correlation, demonstrating that the permeability of a biofilm increases as its number of viable cells decreases. The data are shown in Fig. 4 as a scatterplot.

### Diffusion of Oregon Green into S. mutans biofilm microcolonies.

**FIG 5:**
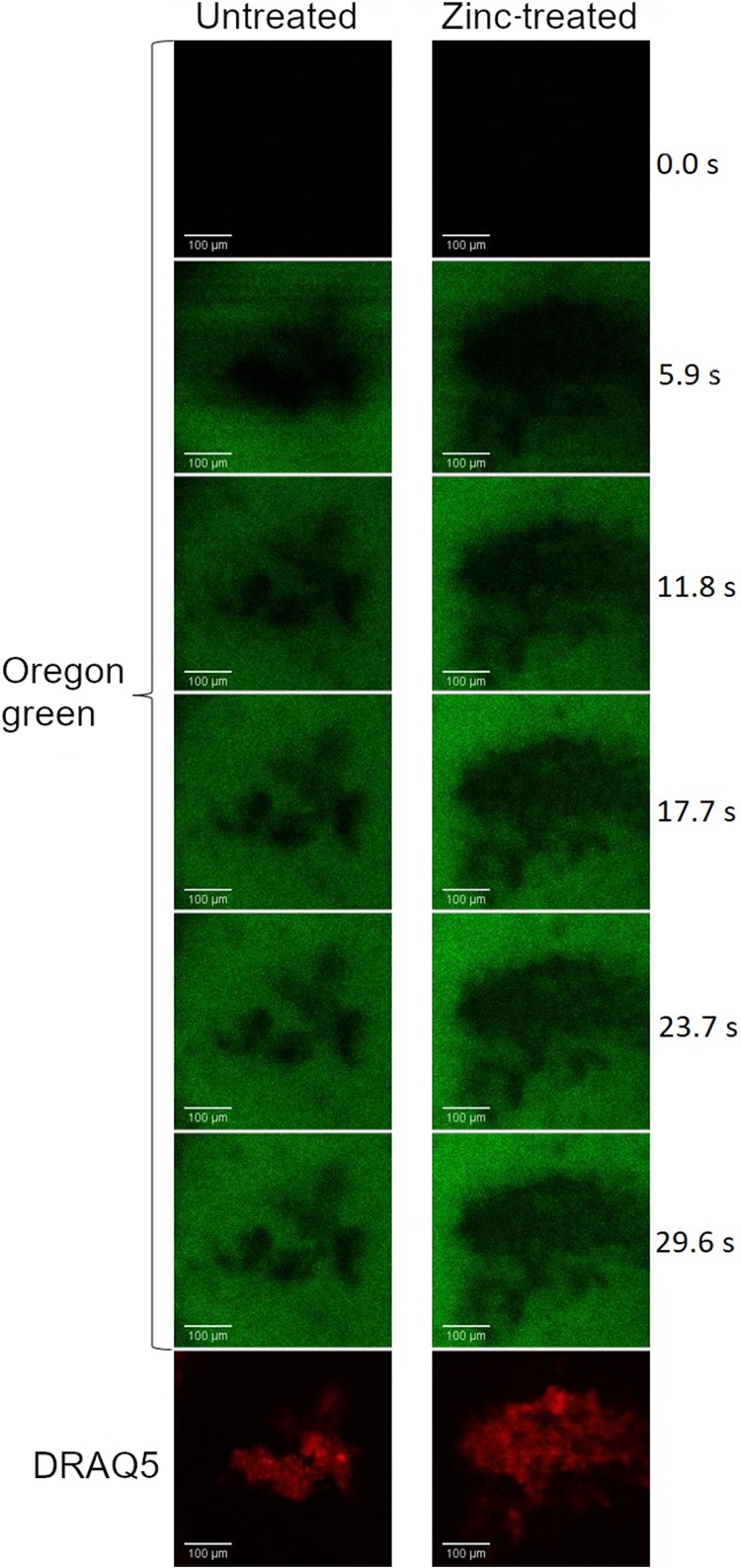
Representative time-series images showing the diffusion of 100 μL of 1 mM Oregon Green through the ibidi channel slide and into the microcolony (nucleic acids stained red with 5 μM DRAQ5). The untreated sample is shown on the left, and the sample treated with 0.25 mg/mL ZA is shown on the right. The scale bar is 100 μm.

**FIG 6:**
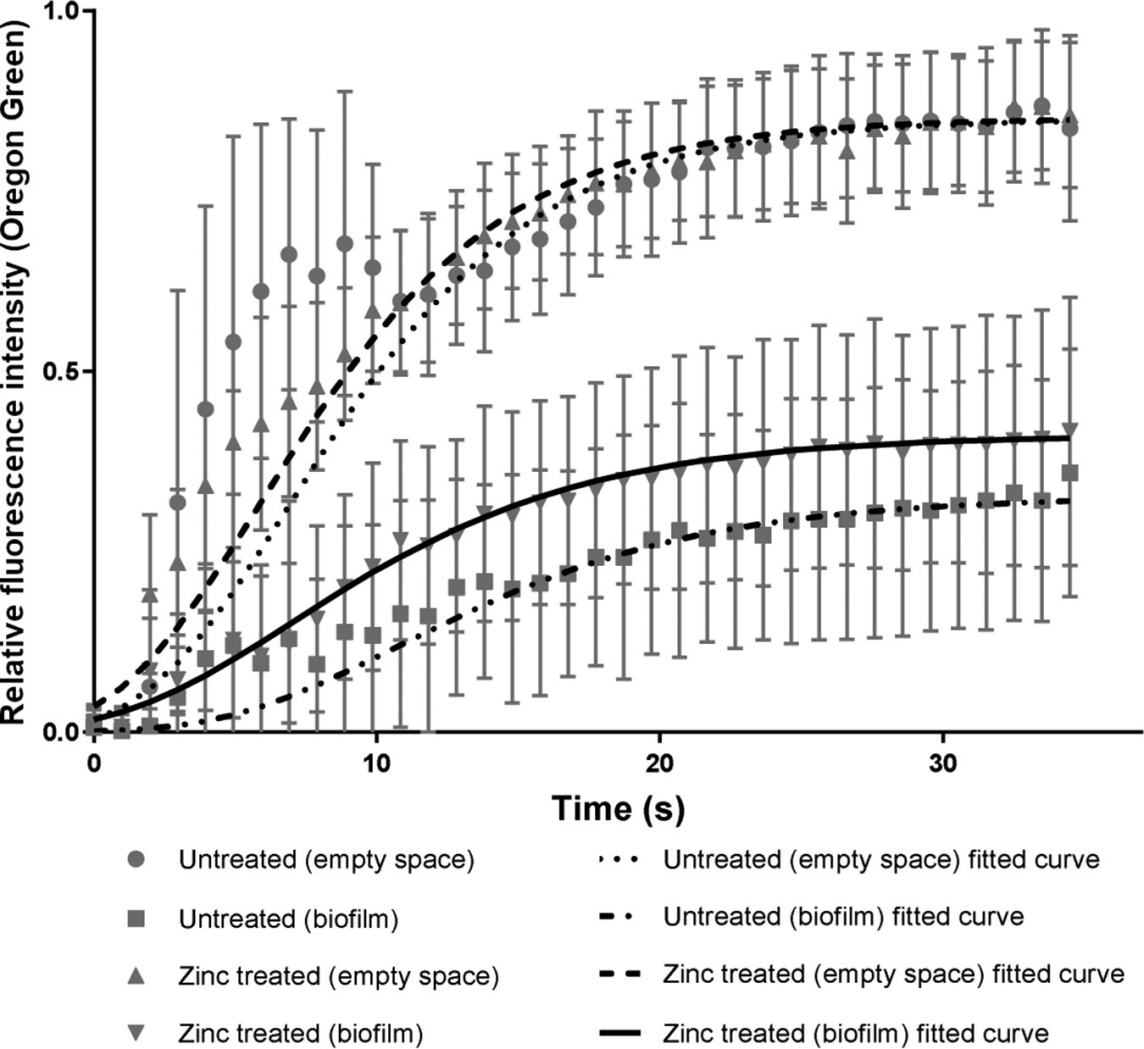
Showing the diffusion of Oregon Green (OG) into the empty space and zinc-treated or untreated S. mutans microcolonies within the ibidi channel slides. The data points represent averages of three biological replicates with four technical repeats each. The curves show models fitted to each series of data points based on the SGompertz predefined function in GraphPad Prism (dotted line = untreated, solid line = zinc treated). The error bars represent the standard error of the mean.

**FIG 7:**
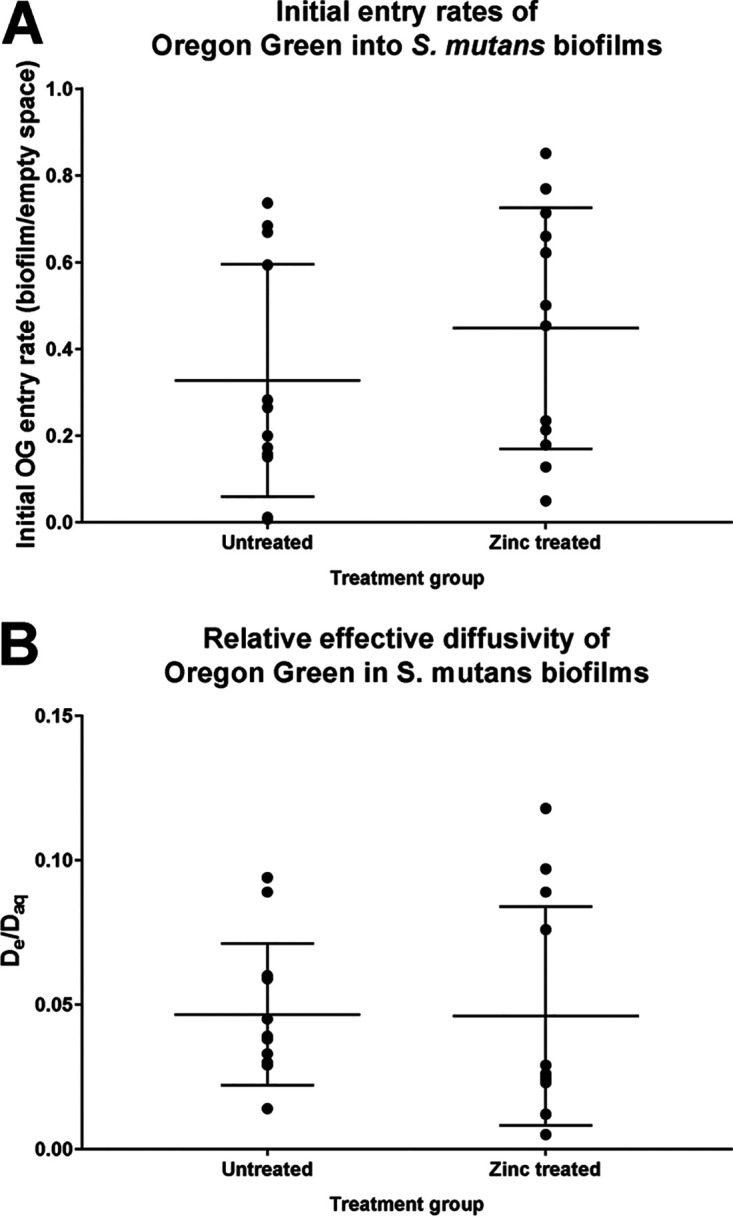
The initial diffusion rates (A) and relative effective diffusivities (B) of OG in zinc-treated and untreated S. mutans biofilms are shown as aligned dot plots, with ● representing individual data points and horizontal lines showing the means and standard deviations. The initial diffusion rate was defined as (slope of OG entering a microcolony) / (slope of OG in empty space) between 2 and 15 s. The relative effective diffusivity was calculated as the ratio between the diffusion coefficient of OG in the biofilm (D_e_) and the diffusion coefficient in water (D_aq_). The values represent three biological replicates with four technical replicates.

To assess the effect of ZA on the permeability of biofilm, a time series capturing the exposure of microcolonies to OG was captured via confocal microscopy. Representative images are shown in Fig. 5. The fluorescent probe entering each biofilm microcolony was enumerated using a SpIDA analysis and fitted using Equation 3.1. Fig. 6 shows the relative fluorescence intensity of OG over time for both the biofilm microcolonies and the empty space within the channels in the zinc-treated and untreated samples. Data points between 2 and 11 s were excluded before curve fitting, due to a transient spike in fluorescence that was associated with the injection of OG into the channel space. The initial biofilm diffusion rates (measured between 2 and 15 s) were calculated as a fraction of the empty space values (±SD) and gave results of 0.45 (±0.28) and 0.33 (±0.27) for the zinc-treated and untreated samples, respectively. The relative effective diffusivity, D_e_/D_aq_, of OG into the microcolony was identical in the zinc-treated and untreated groups, with ratios of 0.049 (±0.043) and 0.049 (±0.026), respectively, and it is similar to the ratio determined by Zhang et al. (33). Their endpoint densities, calculated as an average between frames 34 to 36, were 0.34 (±0.17) and 0.41 (±0.18) relative fluorescence units, respectively. None of these differences were statistically significant, in part due to the substantial biological variation, particularly for the relative effective diffusivities of the zinc-treated samples, as is seen in Fig. 7.

### Coefficients of variation.

The coefficients of variation were calculated for the zinc-treated and untreated samples in the transwell transportation assay, as were the SpIDA-determined initial diffusion rates and relative effective diffusivities. The results are detailed in Table 1, and they show an approximately 2-fold increase in variation for the zinc-treated samples in the transwell transportation assays and in the relative effective diffusivity, compared to those of the untreated samples. The coefficients of variation were high within technical repeats and within the biological replicates for the relative effective diffusion measurements, with values of 82%, 72% and 33% for the zinc-treated samples and 44%, 36% and 26% for the untreated samples for replicates 1, 2, and 3, respectively. There was little difference in the variation of untreated to zinc-treated samples for the initial diffusion rates, with an observed decrease of only 0.24-fold.

**TABLE 1:** The coefficients of variation, SpIDA-determined initial diffusion rates, and relative effective diffusivities were calculated for the transwell transportation assay^*a*^

Expt	Untreated	Zinc-treated		
		0.25 mg/mL	0.5 mg/mL	Fold change
Transwell transportation assay	4%	8%	9%	+2.00/+2.25
Initial diffusion rate	82%	62%	—	−0.24
Relative effective diffusivity	36%	62%	—	+1.72

a The coefficients are the ratios of the standard deviation and the mean of a sample, given as percentages. The fold changes from untreated to zinc-treated samples are given. The data are representative of at least three biological replicates. —, not determined.

## DISCUSSION

Oral bacteria will generally be exposed to diminishing concentrations of compounds that used to control the growth or activities of oral bacteria. The zinc acetate concentrations tested in the current study (0.5 and 0.25 mg/mL) are approximately 50 to 100-fold lower than the concentrations of zinc found in commercially available, zinc-containing dentifrices (2% w/w). If a similar pattern is assumed for zinc, this suggests that the effects observed in the current study may occur in the mouth for several hours post-brushing. For comparison, it has been reported that fluoride remains in the mouth for several hours following brushing, particularly, if there is no consumption of food or drink. Nazzal et al. (34) reported a 3-fold reduction of fluoride concentrations in saliva per hour following brushing with a fluoridated dentifrice. In the current study, the adaption of the transwell transportation assay facilitated the permeation measurements of a fluorescent probe Oregon Green (OG), through S. mutans biofilms in an apical-basolateral direction, illustrating the potential diffusion of dentifrice actives through oral biofilms, down to the tooth surface. This approach revealed a significant increase in the permeability of biofilms grown in the presence of sublethal levels of zinc, compared to untreated controls. At 509 Da, OG is substantially larger than most antimicrobial actives that are added to dentifrices. Thus, in terms of size, an increase in OG diffusion through the biofilm signifies the potential for an increase in the permeability of dentifrice actives. The relationship between the molecular weight (M_W_) of a compound and its diffusion coefficient occurs (fold change in [M_W_]^−1/3^) according to the Stokes-Einstein relationship (32, 35). Therefore, the diffusion of smaller dentifrice actives can be expected to be faster than that observed with OG, and this would also make the absence of an effect due to ZA more obvious. However, other factors, such as charge and hydrophobicity, further affect the diffusion coefficient (36). Zhang et al. (33) reported that the D_e_/D_aq_ of compounds diffusing through an S. mutans biofilm increased as the net charge increased from −2 to +1, suggesting that the opposing charges of a negatively charged compound and the overall negatively charged biofilm decrease diffusion rates. The diffusion of negatively charged fluoride ions may be retarded similarly to OG, which has a net charge of −1 (33).

Changes in the permeability of S. mutans biofilms following 5 days of exposure to sublethal concentrations of zinc acetate were mapped using techniques that investigated the penetration of the biofilm on both the micro and macro scales. The transwell transportation assay is usually applied in eukaryotic cell culture to measure the transport of compounds from the top to the bottom (apical-basolateral direction) or from the bottom to the top (basolateral-apical direction) of cell monolayers (37). We are not aware of its previous use to measure permeability in bacterial studies, but it provides a sensible means of measuring the overall diffusion rate of a compound through mature biofilms.

The increase in transwell biofilm permeability correlated significantly with the number of viable cells present in the biofilm (*P* < 0.05), predictably showing that a decrease in viable count led to an increase in permeability. The disruption of biofilm formation observed between concentrations greater than 0.313 mg/mL ZA further explains this increase in permeability.

Further investigations into biofilm permeability at a microscopic level were conducted using SpIDA, a method first described by Godin et al. (38), which was applied to confocal microscope images to enumerate the number of fluorescent particles in a specified region of interest (ROI). By fitting super-Poissonian curves to intensity histograms that were generated by measuring the fluorescence intensity of specified regions of interest (ROIs) within Confocal laser scanning microscopy (CLSM) images, it is possible to determine the exact number of fluorescent particles present within that space. When measurements are taken at specific intervals over time, it is possible to calculate the diffusion rate of a compound into specific ROIs within microcolonies, thus defining local diffusion patterns.

S. mutans biofilms were cultivated under the same conditions as in the transwell transportation assay in Ibidi channel slides before the influx of OG into microcolonies was quantified. The initial diffusion rates and relative effective diffusivities of OG within microcolonies were calculated, and they showed that although long-term, low-level exposure to zinc slightly increases initial diffusion rates, there were no statistically significant differences between the two treatment groups for initial diffusion, endpoint density, or relative effective diffusivity.

A SpIDA analysis was applied to investigate smaller-scale changes in permeability and also to focus on any initial changes in diffusion with an image time series taken over 34.5 s. While there were no significant changes in initial rates or relative effective diffusivity into the biofilms, there was a marked increase in the endpoint density of the zinc-treated biofilms, and this subsequently developed into a significant change over time, as seen in the transwell transportation assay. There was considerably greater variation in the zinc-treated samples, compared to the untreated controls, in particular, for the transwell transportation assay and relative effective diffusivity.

Although the tested concentrations of zinc did not increase the short-term permeability of the S. mutans microcolonies, they did contribute to an overall increase in permeability that was observable within 1 to 1.5 h by disrupting biofilm formation and increasing heterogeneity. Hence, zinc and other antimicrobial actives may be able to permeate the remaining plaque more effectively for several hours post-brushing.

### Conclusions.

Exposure to sublethal concentrations of zinc over several days increased the overall permeability of S. mutans biofilms. The initial entry rate and diffusion coefficients within small microcolonies were unchanged, but a substantial decrease in biofilm formation coupled with an increase in biofilm heterogeneity led to significant changes in overall biofilm permeability that were observable within 30 min.

## MATERIALS AND METHODS

Tryptone soya broth and Tryptone soya agar (Oxoid, Basingstoke, U.K.) with 2% added sucrose (TSBS and TSAS, respectively) were used to cultivate S. mutans. Fluorescent stains were purchased from Thermo Fisher (Loughborough, U.K.). All other reagents were obtained from Sigma-Aldrich (Gillingham, U.K.), unless otherwise stated.

### Bacterial cultures and sample preparation.

All experiments were performed using the S. mutans NCTC 10449 type strain, which was obtained from Public Health England (PHE, Salisbury, U.K.). Samples of S. mutans were prepared by diluting overnight cultures to a density of approximately 3×10^5^ CFU/mL in TSBS with or without the addition of zinc acetate (ZA, Alfa Aesar, Haysham, U.K.). The growth conditions were anaerobic (10% CO_2_, 10% H_2_, 80% N_2_ gas mixture) at 37°C for all experiments. All results are representative of a minimum of three biological replicates.

### MICs.

The MIC of ZA against S. mutans was determined by following the CLSI broth microdilution guidelines (33). Briefly, stocks of ZA were made in sterile TSBS and serially diluted across 96-well Corning Costar flat-bottomed, polystyrene microtiter plates (Sigma-Aldrich, Dorset, U.K.). Equal volumes of S. mutans cell suspensions (prepared as described above) were added, and the plates were incubated for 24 h before the MIC was determined as the lowest concentration of ZA at which visible growth was inhibited.

### Transwell transportation assay.

A transwell transportation assay protocol, described by Hubatsch et al. (24), was adapted to measure the transport of a 509 Da fluorescent tracer with a net charge of −1 (Oregon Green 488, OG) at set intervals through unfixed S. mutans biofilms that were grown in the presence or absence of ZA in an apical-basolateral direction, so as to mimic the transport of dentifrice actives through dental plaque to a basal surface (teeth). OG has been used previously in the determination of diffusion within S. mutans biofilms and showed good stability across a range of pH values and ionic strengths (33).

**FIG 8:**
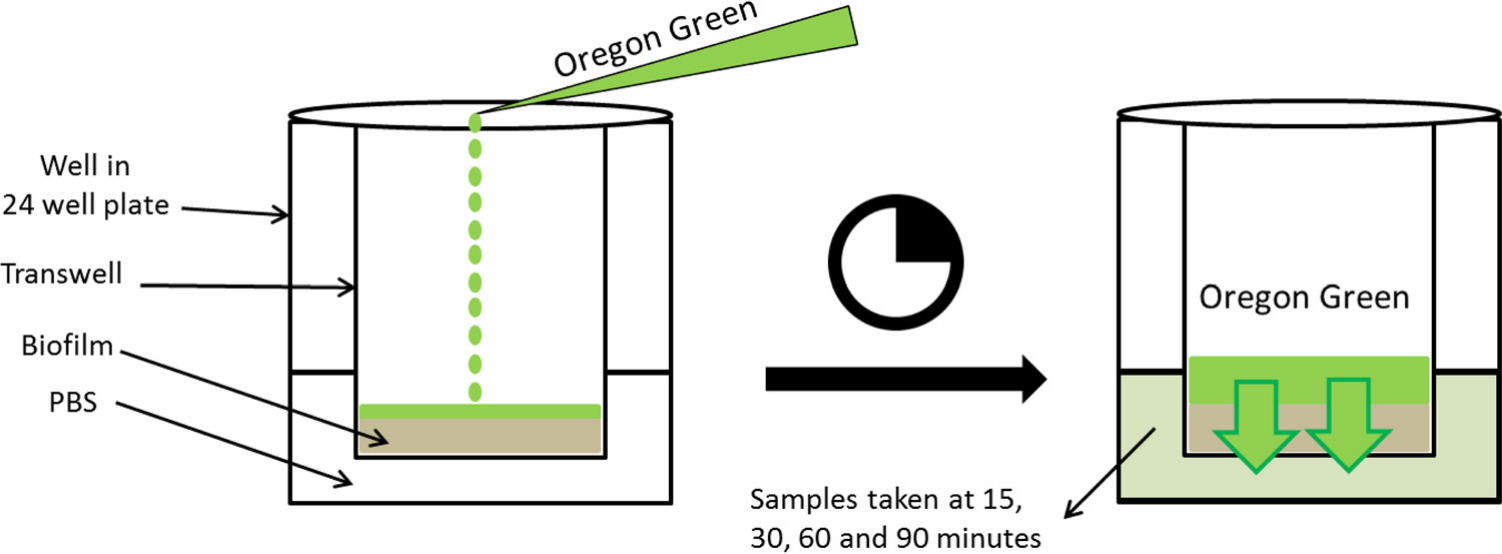
Diagram showing the setup of the transwell transportation assay used to measure the penetration of Oregon Green, a fluorescent tracer, through mature S. mutans biofilms.

Briefly, S. mutans cell suspensions containing 25% or 50% of the MIC of ZA were added to each ThinCert Cell Culture Insert transwell (Greiner Bio-One, Stonehouse, U.K.) and placed in the well of a 24-well Corning Costar flat-bottomed cell culture plate (Sigma-Aldrich, Dorset, U.K.) that contained a further 600 μL of sterile TSBS or TSBS with ZA to match the contents of the transwell. Biofilms were cultivated for 5 d, and the media were changed on days 2 and 4. Following incubation, the medium and any loose cells and debris in the transwell were aspirated via pipette, and the transwell was transferred to a new 24-well culture plate that contained 1 mL of phosphate buffered saline (PBS) per well. 200 μL of 1 mM OG was added to each transwell, and 100 μL samples of PBS were taken from the plate wells at 15, 30, 60 and 90 min, as shown in Fig. 8. The 24-well culture plates were incubated in air at 37°C between samplings. The amount of OG that had moved through the biofilm was quantified using a previously determined standard curve of OG fluorescence intensity and a CLARIOstar microplate plate reader (BMG Labtech Ltd., Aylesbury, U.K., 483 nm excitation, 530 nm emission) that was adjusted to the optimal focal depth and gain using an OG control well (0.5 mM).

The viability of biofilms grown in the presence or absence of 6.25%, 12.5%, 25%, and 50% of the MIC of ZA (i.e., 62.5, 125, 250, and 500 μg/mL) was also determined by suspending the biofilms in half-strength thioglycolate (TG) broth and plating out appropriate dilutions on TSAS for total viable counts. These concentrations were selected to represent the sublethal exposure of bacteria in the oral cavity. Briefly, the basal membrane of each transwell was removed using a scalpel, and the loose membrane and transwell were vortexed for 30 s in 10 mL of TG broth with approximately 1 mL of 3 mm glass beads added. The plates were incubated for 2 d before counting.

### Crystal violet biofilm formation assays.

Crystal violet (CV) biofilm formation assays were performed to determine how the addition of zinc affects the biofilm formation of S. mutans, and the assays were based on the protocol described by O’Toole (39). Briefly, S. mutans cultures were grown in 96-well microtiter plates in the presence of serial dilutions of ZA in TSBS for 24 h with a total well volume of 150 μL. The culture medium and any loose cells were removed following incubation by inverting the plates onto tissue paper before washing them twice via immersion in sterile distilled and deionized water (ddH_2_O). 0.1% (w/v) CV in ddH_2_O was added (200 μL per well) for 30 min before emptying and washing the plates twice and leaving them to dry at room temperature. Once fully dry, 250 μL of absolute ethanol was added for a further hour to solubilize any remaining CV. The absorbance of each well was read at 600 nm using a PowerWave XS plate reader (BioTek, Winooski, USA). Sterile controls were included throughout and were used for background correction.

### Spatial intensity distribution analysis (SpIDA) of confocal images.

**(i) Confocal laser scanning microscopy (CLSM).** Images were captured using a Zeiss LSM 510 inverted confocal microscope (Jena, Germany) with a 20× Plan-Apochromat 0.8 NA objective lens. DRAQ5 was excited using a helium neon laser at 633 nm, and emission was collected using an HFT 514/633 nm beam splitter (BS) and a 650 nm long pass filter. OG was excited using an argon laser at 488 nm, and emission was collected using an HFT 488 nm BS and a 505 to 550 nm band-pass filter. All experiments kept the settings for the laser power, pinhole diameter, and detector and amplifier gain constant.

**FIG 9:**
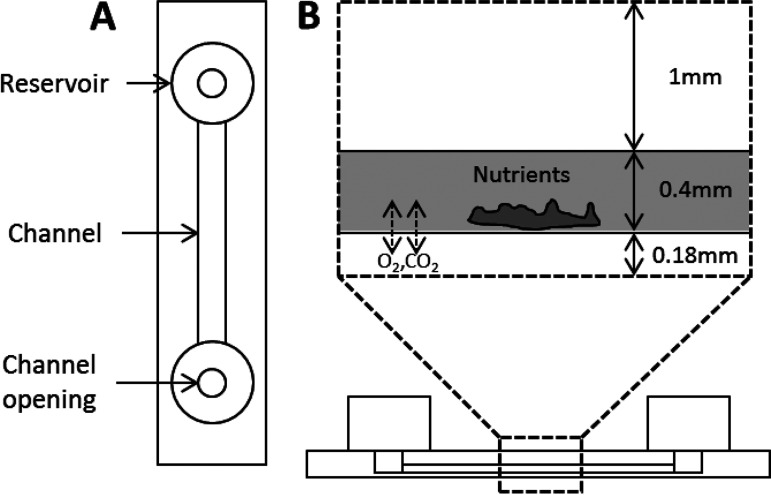
Illustration showing the structure of the Ibidi μ-Slide I channel slide. (A) Top-down view, showing the reservoirs, channel openings, and channel. (B) Side-on view, with an excerpt (in the dashed line box) showing the location and dimensions of the channel within the slide, with gas exchange occurring across the bottom of the channel. Illustration adapted from Ibidi (39).

S. mutans biofilms were cultivated in Ibidi μ-Slide I polymer channel slides (Martinsried, Germany) for 5 d in the presence or absence of 25% of the MIC of ZA before determining the diffusivity of a fluorescent tracer, OG, into the microcolonies. The Ibidi slides, illustrated in Fig. 9, were set up with 100 μL S. mutans cell suspensions in the channel space and 600 μL sterile TSBS in each side reservoir. The media were changed on days 2 and 4.

Following incubation, all broth was removed, and loosely attached cells were removed using 1 mL PBS per, before the remaining cells were stained for 30 min in the dark using 5 μM DRAQ5, which is a red nucleic acid stain. The staining solution was then removed from the channels using filter paper before imaging. One field of view, containing several microcolonies and some presumed empty space (defined by the absence of a visible DRAQ5 signal), was selected per slide, and a time series capturing approximately one 256 × 256 pixel image per second (pixel dwell time of 3.2 μs) was run for 200 frames, with 100 μL of 1 mM OG being injected gradually into the channel over frames 10 to 20.

**(ii) Spatial intensity distribution analysis (SpIDA).** The background and protocol for the SpIDA analysis have been previously described (27).

The image time series were trimmed to include 36 frames, starting 2 frames before the appearance of OG. Eight regions of interest (ROIs) were chosen for each image: four in empty space and four within microcolonies. The density population per beam area (dp/BA) (i.e., the number of fluorescent entities per area) was determined using the protocol described by Godin et al. (38), using the open-access GUI SpIDA software (available for download from the developer’s website: http://www.neurophotonics.ca/en/tools/software). The quantal brightness of OG particles had been previously determined and was entered into the software along with the pixel size and beam size. Corrections for photomultiplier tube (PMT) shot noise and white noise were also entered, both of which were determined by adapting the methods described by Hamrang et al. (40). Briefly, the PMT shot noise was established by measuring the fluorescence intensity (iu) of a densely fluorescent slide of OG (100 μL of 100 mM dried OG) over a range over laser powers. The slope variance (value entered in GUI SpIDA) was calculated by plotting the pixel intensity variation (iu^2^) over the mean pixel intensity (iu). The white noise was calculated by measuring the fluorescence intensity with a black box covering the objective lens. Both measurements were conducted using the GUI SpIDA software package.

### Data analysis.

Unless stated otherwise, all graphs and statistical analyses were generated in GraphPad Prism 7 (La Jolla, CA, USA). The details of each test are provided in greater detail below.

**(i) Transwell transportation assay and viable count correlation.** Data from the transwell transportation assay were plotted as mM of OG over time. The treatment groups were compared using a repeated-measures one-way analysis of variance (ANOVA) with Tukey’s *post hoc* test. The correlation between the viable counts and the biofilm permeability was determined by calculating Pearson’s correlation coefficient. The coefficient of variation was determined for the transwell transportation assay by dividing the standard deviation by the mean.

**(ii) Crystal violet biofilm formation assays.** At each concentration of ZA, the mean absorbance (A_600_) of crystal violet, which is indicative of biofilm quantity, was compared that of the untreated control using a Mann-Whitney test.

**(iii) Spatial intensity distribution analysis (SpIDA).** The monomer and dimer populations were determined for each ROI in each frame, and the total number of fluorescent particles per beam area was plotted over time. Nonlinear curve fitting was performed using the OriginPro software package (OriginLab Corporation, Northampton, MA, U.S.A.), using the SGompertz predefined function:

(3.1) $$y=ae^{-e^{\left(-k\left(x-x_{c}\right)\right)}}$$

where *a* is the amplitude, *x_c_* is the midpoint of the curve, *k* is the growth rate coefficient, and *e* represents Euler’s number (2.718). For fitting purposes, data points were excluded for all of the data sets between frames 4 to 12 due to a spike in fluorescence that was associated with the injection of the OG. The Gompertz model is used in predicting the diffusion of commercial products into the market (36). Therefore, it may not be relevant for the biological diffusion of a compound, but it was applied to facilitate the reading of the SpIDA data.

The effective diffusion coefficient (D_e_) within each microcolony was then determined by manipulating the previously described equation (41) for the determination of the 90% saturation value of a spherical microcolony:

(3.2) $$t_{90}=0.37\frac{R^{2}}{D_{e}}$$

where *R* is the radius of the colony and t_90_ is the time taken to reach 90% of the maximum saturation value for each ROI. The relative effective diffusivity, defined as D_e_/D_aq_, where D_aq_ is the diffusion coefficient of OG in water, was calculated for each ROI. The treatment groups were compared using an unpaired *t* test. The value of D_aq_ for OG has been determined to be 4.1 × 10^−6^ cm^2^ s^−1^ at 25°C (33, 42), and it was adjusted to match the experimental conditions (20°C or 293.15 K) using the following equation:

(2.3) $$D\left(T\right)=D\left(25{}^{\circ}C\right)\times \frac{T}{298.15K}\times \frac{8.9\times 10^{-4}\mathrm{Pa}\times s}{\eta \left(T\right)}$$

where *T* is the temperature in Kelvin, *D* is the diffusion coefficient, and *η* is the viscosity of water, which also changes depending on the temperature and is defined as follows.

(2.4) $$\eta =2.414\times 10^{-5}(\mathrm{Pa}\times s)\times 10^{\frac{247.8K}{T-140K}}$$

The initial diffusion rate of OG (defined as the slopes between 2 and 15 s) was calculated as the ratio of the microcolony diffusion rate over space. The coefficients of variation of the initial diffusion rates and relative effective diffusivities were determined by dividing the standard deviations by the means. The treatment groups were compared using a Mann-Whitney test.
